# Structural and Quantitative Analysis of Polyfluoroalkyl Substances (PFASs) and Para-Phenylenediamines (PPDs) by Direct Analysis in Real Time Ion Mobility Mass Spectrometry (DART-IM-MS)

**DOI:** 10.3390/molecules30132828

**Published:** 2025-06-30

**Authors:** Calum Bochenek, Jack Edwards, Zhibo Liu, Chrys Wesdemiotis

**Affiliations:** 1 Department of Chemistry, The University of Akron, Akron, OH 44325, USA; cb332@uakron.edu (C.B.); je94@uakron.edu (J.E.); 2 School of Polymer Science and Polymer Engineering, The University of Akron, Akron, OH 44325, USA; zl66@uakron.edu

**Keywords:** PFAS, PPD, mass spectrometry, ion mobility, DART ionization

## Abstract

Polyfluoroalkyl substances (PFASs) and para-phenylenediamines (PPDs) are emerging classes of anthropogenic contaminants that are environmentally persistent (most often found in ground and surface water sources), bioaccumulative, and harmful to human health. These chemicals are currently regulated in the US by the Environmental Protection Agency (EPA), the Food and Drug Administration (FDA), and the Occupational Safety and Health Administration (OSHA). Analysis of these contaminants is currently spearheaded by mass spectrometry (MS) coupled to liquid chromatography (LC) because of their high sensitivity and separation capabilities. Although effective, a major flaw in LC-MS analysis is its large consumption of solvents and the amount of time required for each experiment. Direct analysis in real time mass spectrometry (DART-MS) is a new technique that offers high sensitivity and permits rapid analysis with little to no sample preparation. Herein, we present the qualitative and quantitative analysis of PFASs and PPDs by high-resolution DART-MS, interfaced with ion mobility (IM) and tandem mass spectrometry (MS/MS) characterization, demonstrating the utility of this multidimensional approach for the fast separation and detection of environmental contaminants.

## 1. Introduction

“Poly- and perfluoroalkyl substances” (PFASs) is a widely used term that refers to a broad family of chemicals that possess at least one fully fluorinated methyl or methylene group (CF_3−_ or –CF_2−_, respectively) [1]. In most cases, these molecules are extensively fluorinated, with long aliphatic chains saturated with C–F bonds. PFASs have been commonly used in everyday life for almost a century in products such as pharmaceuticals, pesticides, fabrics, nonstick cookware, adhesives, cosmetics, and even food packaging [2,3,4] due to their high stability, water/lipid resistance, and perceived biological inertness [5,6,7]. Although these fluorinated compounds may undergo chemical breakdown in their functional groups (typically carboxylic acid, sulfonic acid, or ether moieties) [8,9], the high energy of the C–F bond (488 kJ/mol) provides exceptional thermodynamic stability and chemical inertness to the polyfluorinated alkyl chain (tail) [10]. The length of the polyfluorinated chain can also influence surfactant properties, with chain lengths of C_4_F_9_–C_16_F_33_ being most common in industrial settings [11,12].

The production of PFASs hit a substantial halt in the early 2000s due to evidence of their environmental persistence and accumulation potential. Specifically, the possible toxicity to humans and animals has led to PFAS becoming an emerging contaminant of concern. The oleophobic and hydrophobic properties of PFASs and widespread application in consumer products has made such compounds omnipresent in the environment, specifically in water [11,12] and air [13,14]. The ability of PFASs to persist in almost any media has endangered humans and animals through the inhalation of such chemicals and the ingestion of food that has been in contact with fluorinated polymers or water contaminated by them [15]. Recent studies have found that high exposure to PFASs can cause diseases such as increased immunosuppression [16], ulcerative colitis [17], elevated cholesterol [18], and even cancer [19]. For these reasons, health organizations around the world have introduced restrictions on the amount of PFASs allowed in human products. For example, health guidelines by the Australian Department of Health state that a tolerable daily intake (TDI) of 0.16 and 0.02 μg/kg/d for perfluorooctanoic acid (PFOA) and perfluorooctanesulfonic acid (PFOS), respectively, is acceptable [20]. Meanwhile, the European Union listed PFASs as compounds for international regulatory consideration in the 2010s [21], with the Environmental Protection Agency (EPA) and World Health Organization (WHO) quickly following soon after [9]. This led to a restriction for the total allowable concentration of some PFAS chemicals in the United States to <70 ng/L in 2016, followed by a more drastic restriction in 2022 for specific, longer-chain PFASs such as PFOA and PFOS to 0.004 ng/L and to 0.02 ng/L, respectively [12,14,22]. These regulations have resulted in a reduction in PFAS production, especially of longer-chain PFAS compounds. While long chain PFAS production continues in emerging economies, many industries have started to explore the use of shorter-chain PFASs, like perfluoropenanoic acid (PFPA), as they tend to be less bioaccumulative.

Another class of environmental contaminants that can cause detrimental health effects are para-phenylenediamine (PPD) antioxidant additives in commercial materials, such as automobile tires. PPDs are significant environmental pollutants because of their toxicity, persistence, and potential to form harmful byproducts [23]. Widely used in hair dyes, textile manufacturing, and rubber products, PPDs frequently enter the environment through wastewater discharge, posing a serious threat to aquatic life. Even at low concentrations, PPDs are highly toxic to fish, invertebrates, and algae, leading to oxidative stress, hematological toxicity, and the disruption of cellular functions [24,25,26]. Furthermore, PPDs can bioaccumulate in aquatic organisms, increasing the risk of toxicity at higher levels of the food chain. Their environmental persistence is particularly concerning, as PPDs do not easily degrade under natural conditions [23]. Instead, they undergo oxidation into reactive quinone compounds, such as 6-PPD-Q, which are even more toxic and contribute to long-term water and soil contamination. In terrestrial environments, PPDs bind strongly to soil particles, thus becoming immobilized and difficult to remove, which leads to prolonged contamination in the affected areas. Moreover, PPDs can leach into groundwater, contaminating drinking water supplies and posing potential health risks to humans [27]. PPDs in agricultural irrigation water can also inhibit seed germination, stunt root development, and negatively impact soil-dwelling organisms such as earthworms and beneficial microbes, which are essential for maintaining soil fertility and ecosystem balance [28]. PPDs can react with other environmental chemicals to form dangerous byproducts, including aromatic amines, N-nitrosamines, and chlorinated derivatives, many of which are known carcinogens or highly toxic compounds [29,30,31]. This is particularly concerning in water treatment facilities, where PPDs can interact with chlorine to form harmful byproducts, further complicating water purification efforts [31]. Given these severe environmental issues, there is an ever-growing need for analytical techniques to help and support decision making at these levels.

Analytical techniques for the identification and characterization of PFASs and PPDs are essential to understanding their effects in both the environment and human health, as well as promoting remedies to these problems. Unfortunately, PFASs and PPDs have become increasingly complex as new alternatives and replacement chemicals are being developed, posing unique challenges to their analysis. In the last ten years, a plethora of diverse identification strategies have been investigated, allowing for the establishment of sample preparation and reliable quantitative methods for the characterization of these types of chemicals. Techniques such as gas chromatography (GC) [32], nanoparticle sensors [33], total oxidizable precursors assay (TOPA) [34], fluorine-19 nuclear magnetic resonance spectroscopy (19F-NMR) [35], and X-ray photoelectron spectroscopy (XPS) [36] have been successfully used. The gold standard for the analysis of PFASs and PPDs has been liquid chromatography mass spectrometry (LC-MS) due to its high sensitivity, effective separation of isomeric species, and quantification potential over a wide range of concentrations [37].

Ion mobility mass spectrometry (IM-MS) has recently emerged as a common alternative to LC because of its ability to separate ions, including isomeric species, by their size, shape, and charge state. IM separation is based on the ions’ transport time through a carrier gas in the presence of an electric field [38]. The IM technology originates from early drift tube experiments, wherein a constant axial electric field propels ions through a region filled with carrier gas. Ions traveling through this region are successively detected based on their migration time through the gas (drift time), which is directly correlated with the ions’ size and shape, as defined by the corresponding collision cross section (CCS). The CCS is commonly reported in units of square angstroms (Å^2^) and describes the averaged forward-moving area of the ion as it drifts through the buffer gas. This concept differs from other, conventional separation techniques that have been linked to MS, such as GC and LC, which disperse samples based on differences in polarity and volatility. The latter techniques are often laborious and time-consuming (several minutes to hours per GC or LC run), whereas ion mobility separations are incredibly fast (<100 ms per spectrum), which is ideally suitable for high-throughput screening workflows. Different variations in IM platforms exist, including drift tube ion mobility spectrometry (DTIMS), traveling wave ion mobility spectrometry (TWIMS), and trapped ion mobility spectrometry (TIMS), which are currently available on Agilent, Waters, and Bruker mass spectrometers, respectively [39]. Our study utilized TIMS, one of the newest IM platforms, characterized by high mobility resolution (>250) along with high IM-MS peak capacity and CCS accuracy [40]. In TIMS experiments, ions are “trapped” by an axial electric field that opposes the forward pushing force of the carrier gas. In contrast, DTIMS and TWIMS employ a forward pushing field that counteracts the drag force of the carrier gas [39]. Because of this difference, ions of smaller size (smaller CCS) are eluted first in DTIMS and TWIMS but last in TIMS, whereas the reverse elution order applies to larger sized ions.

While using the IM-MS platform instead of GC-MS or LC-MS significantly reduces analysis time per sample, an additional increase in sample throughput can be achieved by employing an ambient ionization technique with minimal or no sample preparation requirements. Direct analysis in real-time mass spectrometry (DART-MS) fulfills this prerequisite [41], further facilitating high-throughput screening in both positive and negative ion modes. This is beneficial for PFAS analysis, as over 9000 such chemicals are listed in EPA’s master list of PFAS substances [7]. The utility of DART-MS for PFAS detection has been confirmed in recent publications [41,42]. However, no study so far has focused on the characterization and quantification of PFASs and PPDs using DART ionization alongside IM-MS and tandem mass spectrometry (MS/MS). Here, we demonstrate that high-resolution DART-IM-MS and MS/MS can be used to separate and detect such environmental contaminants using little sample preparation and high-throughput. This approach has the potential to overcome key challenges associated with current PFAS or PPD analysis techniques that are more time-consuming and require large amounts of solvents and auxiliary materials for each experiment.

DART-IM-MS experiments are completed within seconds and have minimal solvent requirements [41]. In contrast, LC-MS experiments take several minutes to an hour and require chromatography-grade solvents, as well as tedious sample preparation procedures to create analyte solutions with concentrations that do not overload or harm the column and mobile phase. As a result of these differences, DART-IM-MS is the more economic choice and ideally suitable for rapid screening and high-throughput applications, provided the IM dimension offers the desired separation efficiency.

PFASs, PPDs, and other environmental contaminants are usually present within complex matrices, like soil, water, or biological specimens. The analysis of such samples poses several challenges due to the compositional complexity of the matrices and the chemical properties of the contaminants. Ionization suppression and isobaric/isomeric interferences by other substances in the sample complicate confident and accurate identification and quantitation. Moreover, PFAS and PPD chemicals can be adsorbed onto surfaces, leading to sample loss, and may be derivatized in the environment, necessitating non-targeted analysis. To address these challenges, a non-discriminatory sample preparation method and a sensitive analytical technique are imperative. DART-IM-MS provides these advantages by minimizing or avoiding solvent use, which reduces the probability of analyte suppression, while offering two-dimensional dispersion, using mass and ion mobility, to resolve transformed substances and overlapping isomers/isobars/conformers, respectively, so that precise and conclusive identification of the desired analyte is achieved. These capabilities will be demonstrated with the investigation of a real-world sample, involving the detection and quantitation of a PPD antioxidant in automobile tires.

## 2. Results and Discussion

### 2.1. DART Quantitation of PFASs and PPDs

The PFAS substances investigated include perfluoropentanoic acid (PFPA), perfluorohexanoic acid (PFHexA), and perfluoroheptanoic acid (PFHeptA); cf. Figure S1. All give rise to deprotonated and decarboxylated ions in negative DART-MS mode, viz., [M − H]^−^ and [M − CO_2_H]^−^, respectively. The PPDs investigated include N-cyclohexyl-N’-phenyl-p-phenylenediamine (C-PPD), N-(1,3-dimethyl)-N’-phenyl-p-phenylenediamine (6-PPD), and 6-PPD quinone (6-PPD-Q); cf. Figure S2. All produce protonated and ammoniated ions in positive DART-MS mode, viz., [M + H]^+^ and [M + NH_4_]^+^, respectively.

To obtain the highest sensitivity possible, the DART source was optimized by adjusting the carrier gas temperature, linear rail speed of the sample holder, electric grid voltage, and vacuum pressure (see below and Materials and Methods section). Helium (He) was selected as the carrier/ionization gas over other less expensive gases (N_2_, Ar, or Ne) because of the high energy of its electronically excited (“metastable”) state (19.8 eV) [41]. The other carrier gases become less energetically excited under DART conditions, which significantly reduces the analyte ionization efficiency. The energy provided by the metastable He was essential for the detection of low PFAS or PPD concentrations. On the other hand, carrier gases such as N_2_, Ar, or Ne can reduce background noise from adventitious molecules present in ambient air (cf. Figure S3); fortunately, these background ions can serve as internal calibrants for accurate mass measurement and elemental composition confirmation of the PFAS and PPD species.

For the PFASs examined, the relative intensity of the [M − CO_2_H]^−^ ion was consistently higher than that of the corresponding [M − H]^−^ ion under the DART conditions used; therefore, [M − CO_2_H]^−^ was selected for quantitation. For the PPDs on the other hand, the [M + H]^+^ ion, which was more abundant than the corresponding [M + NH_4_]^+^ ion, was used for quantitative analysis. The grid voltage of the DART source was varied from 50 to 350 V, the He gas temperature from 150 to 500 °C, and the linear rail speed of the sample holder (Figure S4) between 0.2 and 1.0 mm/s. At each setting of these parameters, experiments were run in triplicate, with the average peak intensity being recorded to determine the optimal conditions for quantitation. The results revealed optimal responses at a temperature of 250 °C, a linear rail speed of 0.5 mm/s, and an electric grid voltage of 150 V.

**Figure 1:**
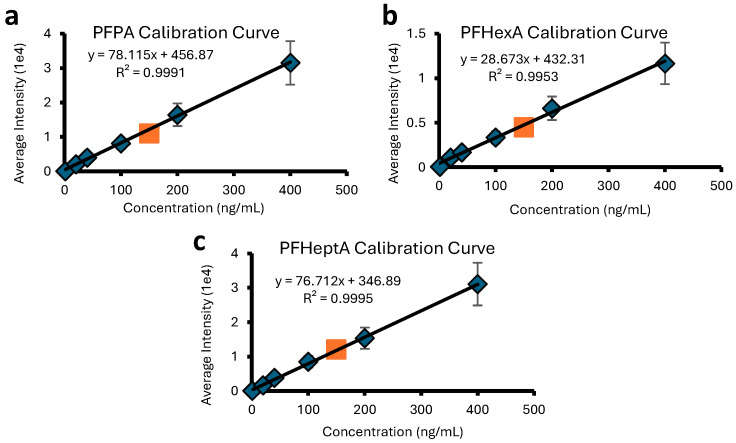
Calibration curves for the quantitation of PFAS compounds, (**a**) PFPA, (**b**) PFHexA, and (**c**) PFHeptA, using [M − CO_2_H]^−^ peak intensities. Blue diamonds correspond to standard solutions with concentrations within 1–400 ng/mL, and the orange square corresponds to a sample at 150 ng/mL treated as an “unknown” for which DART-MS renders 147 ng/mL (within ±2%).

**Figure 2:**
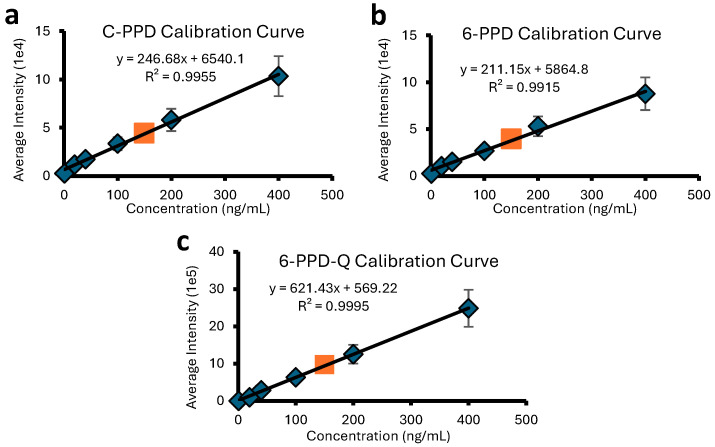
Calibration curves for the quantitation of PPD compounds, (**a**) C-PPD, (**b**) 6-PPD, and (**c**) 6-PPD-Q, using [M + H]^+^ peak intensities. Blue diamonds correspond to standard solutions with concentrations within 1–400 ng/mL, and the orange square corresponds to a sample at 150 ng/mL treated as an “unknown” for which DART-MS renders 143 ng/mL (within ±5%).

To generate calibration curves, PFAS and PPD samples were dissolved in LC-MS-grade ultrapure water and diluted to concentrations of 1, 20, 40, 100, 200 and 400 ng/mL. These samples were analyzed using a DART SVP JumpShot ionization source (Figure S5) coupled to a Bruker timsTOF Pro2 quadrupole/time-of-flight (Q/ToF) mass spectrometer. Each sample was measured in triplicate, with an “unknown” sample at a concentration of 150 ng/mL being run on different days to confirm the accuracy of the curve. The calibration curves for the different PFAS molecules are shown in Figure 1. The results for perfluoropentanoic, perfluorohexanoic, and perfluoroheptanoic acid show excellent linear correlations for measured peak intensity vs. PFAS concentration. Very similar concentration ranges can be quantified for the three investigated PFASs, with the lowest quantifiable amount being ~1 ng/mL (1 ppb) for all samples. The calibration curves for the three investigated PPD molecules also exhibit excellent correlation coefficients (cf. Figure 2) over similar concentration ranges and the lowest quantifiable amounts (1 ppb).

The calibration curves in Figure 1 and Figure 2 do not pass exactly through the (0,0) origin. This deviation is attributed to instrumental factors, specifically background electronic noise during spectral acquisition. The limits of quantification (LOQ) for each PFAS and PPD were calculated based on the lowest point in each calibration curve. Unknown quantities could be determined with an accuracy of greater than ±5% (see legends of Figure 1 and Figure 2).

### 2.2. DART-MS/MS Analysis of PFASs and PPDs

**Figure 3:**
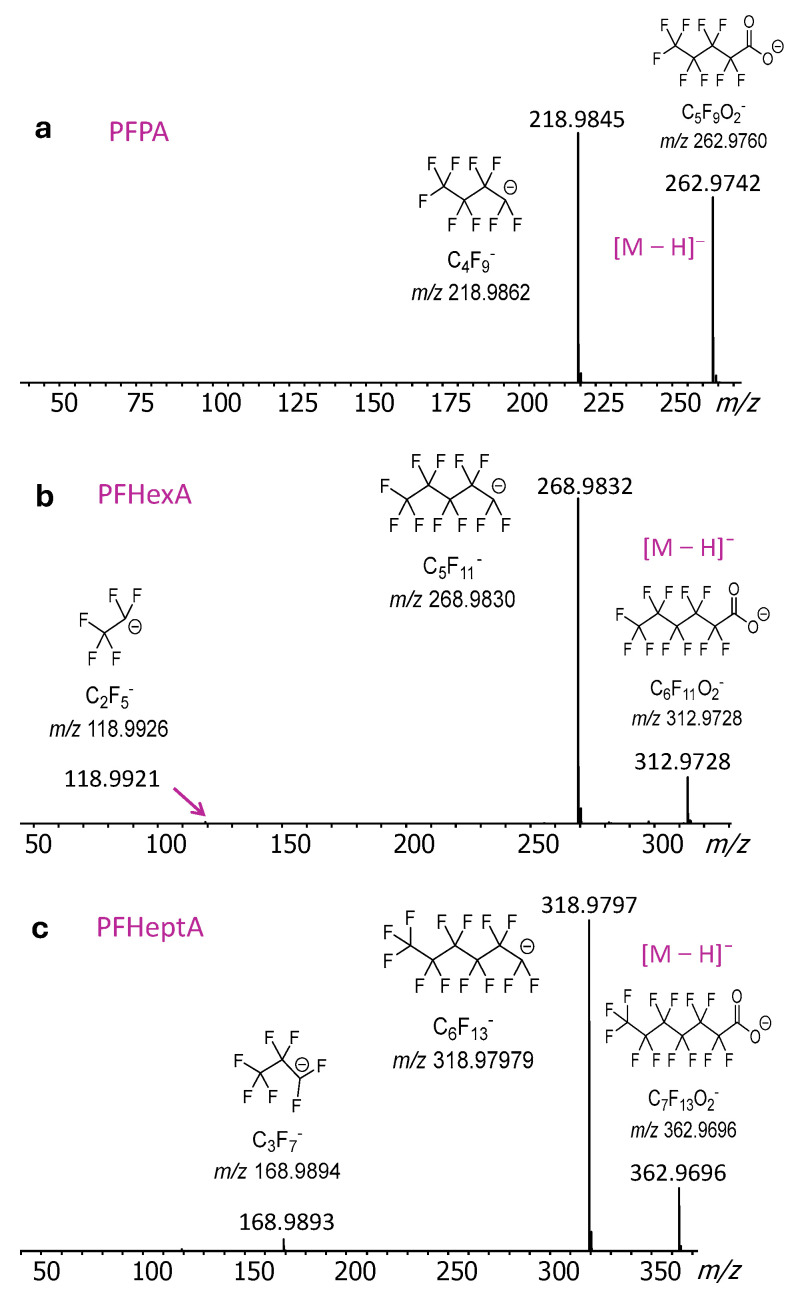
DART-MS/MS spectra of the [M − H]^−^ ions from PFAS compounds (**a**) PFPA, (**b**) PFHexA, and (**c**) PFHeptA. Measured monoisotopic *m*/*z* values are given on top of the peaks and calculated monoisotopic *m*/*z* values are marked under the elemental compositions.

Fragmentation is critical for identifying and characterizing the corresponding molecular structure, as it provides detailed substructure and substituent information that can help to distinguish compounds with identical or very similar molecular weights (isomers and isobars, respectively). This is especially important for PFASs, for which both isomeric as well as isobaric molecules exist that can be differentiated based on their fragmentation patterns. Deprotonated poly/perfluorinated carboxylic acids dissociate through unique fragmentation pathways, initiated by decarboxylation (CO_2_ loss) at the ionized head followed by bond cleavages in the backbone, commonly referred to as an “unzipping” process (C_3_F_6_ losses). This reactivity creates a series of fluorinated fragments (C_3_F_7_^−^, C_4_F_9_^−^, etc.) depending on the length of the chain. The MS/MS spectra of the three PFAS molecules examined (Figure 3) affirm these fragmentation features.

**Figure 4:**
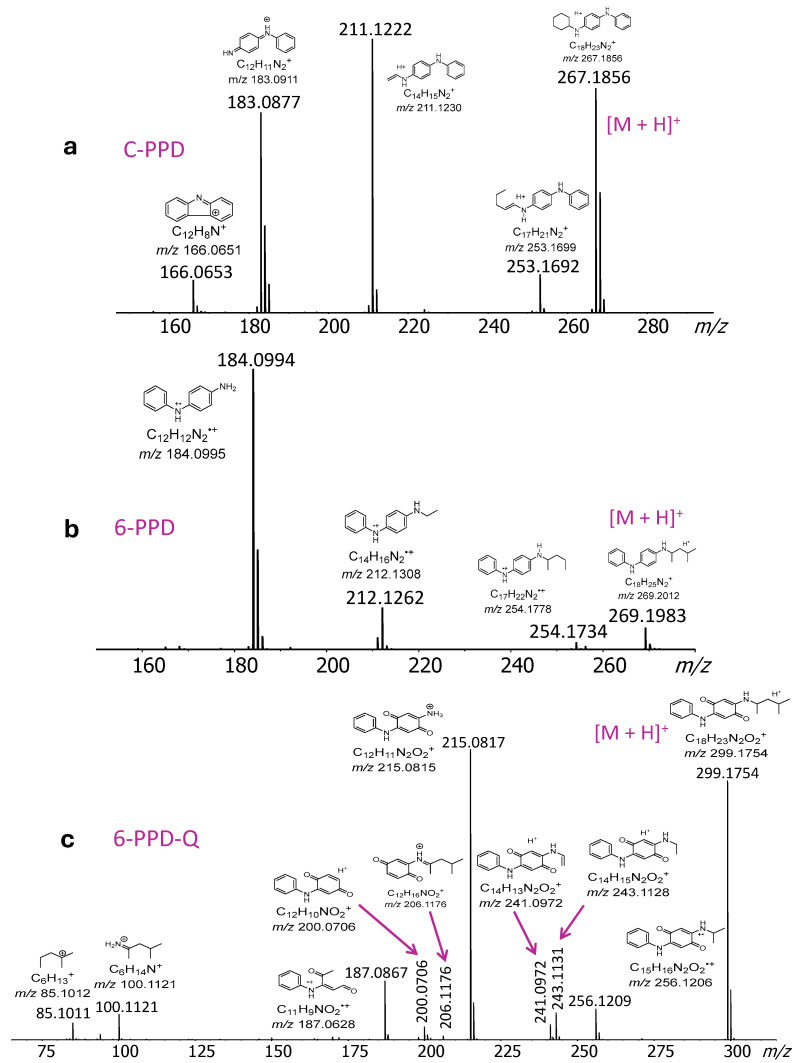
DART-MS/MS spectra of the [M + H]^+^ ions from PPD compounds (**a**) C-PPD, (**b**) 6-PPD, and (**c**) 6-PPD-Q. Measured monoisotopic *m*/*z* values are given on top of the peaks and calculated monoisotopic *m*/*z* values are marked under the elemental compositions.

Conversely, the protonated PPDs undergo extensive fragmentation at their substituent sites, generating fragments that retain the p-phenylenediamine substructure, which readily retains the charge due to its high gas phase basicity and low ionization energy; cf. Figure 4. Interestingly, radical ion fragments are also observed, reflecting the low ionization energy of amines and the stabilization of radical sites in highly conjugated systems.

Several DART ion source parameters can critically influence the quality of MS and MS/MS spectra. Based on our MS/MS study, the carrier gas generating the plasma components that strike and ionize the sample has the most profound effect, with He maximizing ion intensities to allow for MS/MS analysis. Using a carrier gas that reaches lower excitation levels, like N_2_, can reduce in-source fragmentation, but it can also significantly reduce the ionization efficiency of the analyte to the point that MS/MS experiments become intractable. The plasma heater temperature is another important determinant for the ionization efficiency, fragmentation, and decay of analytes. Generally, higher temperatures enhance ion yields by increasing the energy available to promote the desorption and gas-phase ionization; however, too high of a temperature may lead to excessive in-source fragmentation or thermal degradation of sensitive compounds. Conversely, lowering the plasma temperature too much may lead to ineffective desorption, reduced ionization efficiency, and lower sensitivity. The electric grid voltage also plays a significant role in ionization efficiency as well as ion transmission. This voltage affects the movement and kinetic energy of the ions as they exit the ion source, allowing for optimization of the balance between molecular ion preservation and in-source fragmentation. Higher grid voltages tend to enhance ionization by accelerating ions toward the mass spectrometer, leading to increased signal intensity. However, if the voltage is too high, it may lead to excessive ion losses and reduced sensitivity. For optimal results, it is better to apply as low of a grid voltage as possible without sacrificing ionization efficiency. Low grid voltages are especially useful for detecting high-mass or thermally labile compounds such as PFASs.

### 2.3. DART-IM-MS Analysis of PFAS and PPDs

A major aim of this study was to determine the collision cross sections of known PFAS and PPD compounds and their fragments to enrich the database of available CCS data on environmental contaminants. As stated previously, there are several different types of ion mobility spectrometry that have been coupled to mass spectrometers. The TIMS variant utilized in our study does not allow for MS/MS fragmentation on mass-selected ions prior to mobility separation. This problem can be bypassed by collisionally activating inside the TIMS region during the ion accumulation step, before the TIMS potential is ramped for IM analysis [43,44] (vide infra). Using this approach makes it possible to derive CCS data on fragments. Fragmentation or thermal degradation of the molecular species of interest may already occur spontaneously in the DART ion source, which also enables the derivation of fragment ion CCS data.

**Figure 5:**
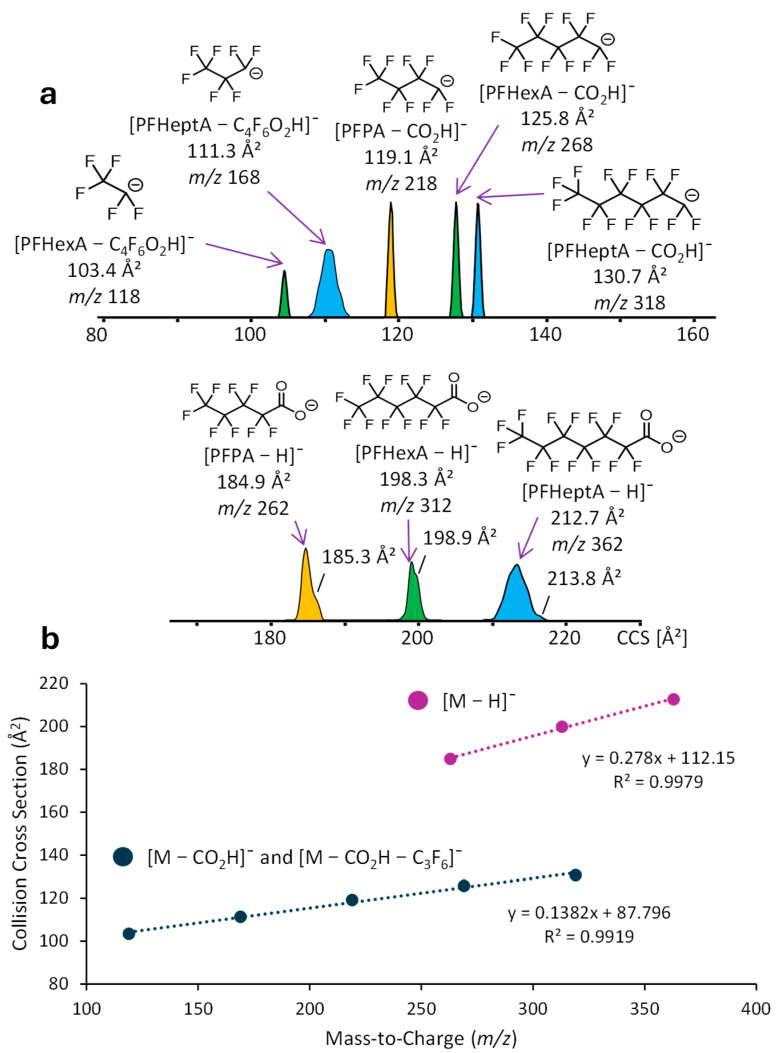
(**a**) Collision cross sections (CCS in Å^2^) of the [M − H]^−^ ions from the PFAS molecules, PFPA, PFHexA, and PFHeptA, and their fragment ions. (**b**) Graph showing linear relationships between CCS values of the various PFAS ions. The apexes of the CCS distributions were used to construct these trendlines.

**Figure 6:**
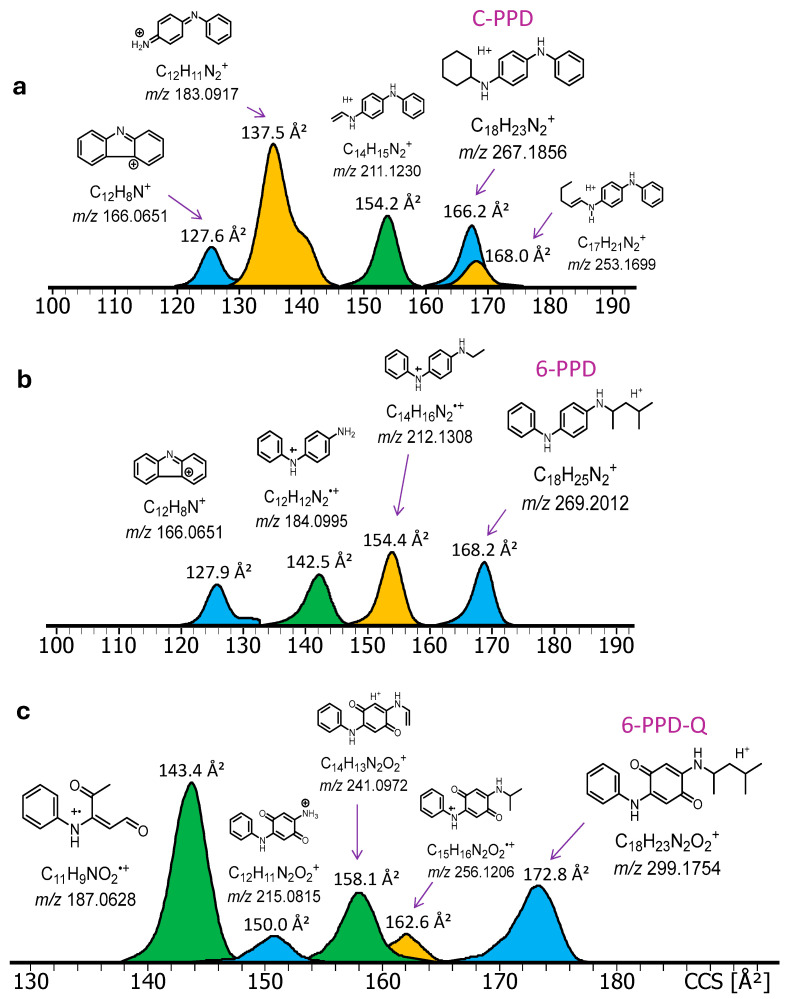
Collision cross sections (CCS in Å^2^) of the [M + H]^+^ ions from the PPD molecules and their fragments. (**a**) C-PPD and fragments; (**b**) 6-PPD and fragments; (**c**) C-PPD-Q and fragments.

To assess how well IM-MS can resolve and separate PFASs, PPDs, and their fragments, all ions in the acquired DART-MS spectra with a signal-to-noise ratio of >5 were mobility separated to determine their CCS. To maximize the sensitivity and fragmentation efficiency, the DART source temperature was set to 250 °C, the electric grid voltage to 100 V, and the linear rail speed of the sample holder to 0.5 mm/s. IM-MS analysis was performed with collisional activation in the TIMS region to obtain CCS data for molecular species as well as fragment ions (see Section 3.3.1 for details). The results are summarized in Figure 5 and Figure 6, respectively.

For the PFAS compounds, the CCS increases linearly with mass (*m*) for the [M − H]^−^ ions and their fragments (cf. Figure 5). The slope of the corresponding trendlines, CCS/*m*, which describes the packing density in the ion structures, is twice as high for the deprotonated species as compared to their fragments, consistent with a decrease in packing density (lower compactness) in the carboxylate terminated chains. Perfluorinated alkyl chains are known to adopt helical conformations [45,46,47,48,49], which can vary in compactness [46] and depend on the substituents and environment of the perfluorinated chain [48,49], justifying the different CCS trends between deprotonated and decarboxylated PFAS ions. It is worth noting that the [M − H]^−^ ions from PFPA and PFHexA exhibit composite mobility distributions with shoulders at higher CCS; while a much smaller shoulder at higher CCS and a broader overall CCS distribution is observed for PFHeptA. This finding suggests that a fraction of the [M − H]^−^ ions may have random coil conformation, which is less compact than helices [50], or a different helical conformation with lower compactness (higher packing density) [46]. It is also observed that the CCS distributions (mobilogram peaks) vary in broadness, as defined by the corresponding full width at half maximum, with those for PFHeptA (*m*/*z* 362) and its fragment at *m*/*z* 168 being noticeably wider. This variability is ascribed to differences in the conformational flexibility of such polyfluorinated anions.

The results of the IM-MS analysis on C-PPD, 6-PPD, and 6-PPD-Q can be seen in Figure 6. Expectedly, protonated C-PPD exhibits a smaller CCS than protonated 6-PPD, reflecting the higher compactness of a cyclohexyl vs. a branched C_6_H_13_ group attached to the PPD connectivity. Another noticeable difference is between the closed-shell fragment at *m*/*z* 183 (from C-PPD) and the radical ion at *m*/*z* 184 (from 6-PPD). The latter fragment is more extended to accommodate charge and radical sites, whereas the closed-shell ion attains two different conformations, one similar to the radical ion and one more compact, probably containing a bent phenyl ring. It is worth noting that an ethyl vs. an ethylene substituent do not change measurably molecular size, as attested by the similar CCSs of *m*/*z* 211 (from C-PPD) and 212 (from 6-PPD). Finally, the tailing of the CCS distributions towards lower CCS suggests that a small population of these PPDs can attain a more compact (folded) structure. For the fragments, the tailing may also result from slight defocusing upon collisional activation in the TIMS region during the accumulation step [43,44].

### 2.4. DART-IM-MS Analysis of 6-PPD in Tire Treads

The applicability of the DART-IM-MS method to real-world samples was tested with the quantitative analysis of 6-PPD in a used Hankook automobile tire. This paraphenylenediamine is used in tire manufacturing as an antioxidant and antiozonant to prevent degradation. In new tires, 6-PPD is present at concentrations within 0.4–2 mass%, but this value varies among different parts of the tire and decreases over time due to loss to the environment [51].

For our quantitation study, a 10 mg cut from the tire tread was immersed in 100 μL of tetrahydrofuran (THF) for approximately 7 days. The extract was analyzed following the procedure used for the PPD standards (vide supra). The acquired DART-MS spectrum is shown in Figure S6. It contains a peak for protonated 6-PPD (*m*/*z* 269) with an intensity that corresponds to a concentration of 43.94 ng/mL based on the calibration curve of Figure 2b. Normalization to the mass of the tire piece reveals that 0.44 μg of 6-PPD were released per gram tire tread into the solvent over the 7-day extraction period. These results provide evidence that DART-MS and DART-IM-MS are promising approaches for the rapid determination of antioxidants/antiozonants and other environmental contaminants in complex matrices.

## 3. Materials and Methods

### 3.1. Materials

Methanol and water, both Optima LC-MS grade, were purchased from Fisher Scientific (Fair Lawn, NJ, USA). PFAS and PPD standards were purchased from Sigma Aldrich (St. Louis, MO, USA). The calibrant solution for both positive and negative mode mass calibration as well as reduced mobility calibration for CCS determination was ESI-L Low Concentration Tuning Mix (Tune Mix), acquired from Agilent Technologies (Santa Clara, CA, USA). The Tune Mix components, the ions used for calibration, their elemental compositions and monoisotopic *m*/*z* values, and their reduced mobilities (1/K_0_) are provided in Figure S7.

### 3.2. Preparation of PFAS and PPD Samples and Internal Standards

The PFAS and PPD samples studied were prepared by a series of dilutions from a 10 mg/mL stock solution in water, made in a micro centrifuge tube and mixed by vortex. QuickStrips (Figure S4), which are 12-spot wire mesh cards obtained from Bruker (Billerica, MA, USA), were used to introduce the samples to the DART ionization source. The diluted solutions were spotted on the QuickStrip cards starting with a blank spot, then moved from low (1 ng/mL) to high (400 ng/mL) concentrations. Two blanks were left directly after the 400 ng/mL spot, and the solution with “unknown” concentration was spotted on the last spot.

### 3.3. Instrumentation and Software

#### 3.3.1. Mass Spectrometer

DART-MS, MS/MS, and IM-MS experiments were performed on a timsTOF Pro 2, Q/ToF mass spectrometer (Bruker, Billerica, MA, USA), which is equipped with a TIMS device in front of the Q mass analyzer. All measurements were performed in triplicate. The mass range probed was *m*/*z* 50–1200, and the mass resolving power at *m*/*z* 322 was 32,200 full width at half maximum (FWHM). The polarity of the timsTOF was set to either positive or negative mode depending on the ions of interest. For MS/MS, precursor ions were selected by Q with an isolation window of ±3 Da, and the collision voltage was set between 10 and 40 eV depending on the ion of interest. Trapped ion mobility experiments were carried out by calibrating the ion mobility (and CCS) scale with Agilent tune mix. For the CCS values of molecular species and fragment ions, mobilograms were collected with the voltage between the entrance funnel and TIMS analyzer (Δ6) set to 150 V. The average centroid of each fitted mobility peak is reported, unless noted otherwise. All CCS data are ^TIMS^CCS_N2_ values based on a recent nomenclature recommendation [52].

#### 3.3.2. DART Ionization Source Parameters

The experiments were performed using a Bruker DART-SVP JumpShot ionization source (Billerica, MA, USA), which was coupled to the mass spectrometer through the VAPUR interface pictured in Figure S5. The distance between the DART source and the transfer capillary (ion entrance into the mass spectrometer) was set to 8.5 cm, and ionization mode was set to negative for the PFAS and positive for the PPD samples. The ionization gas was either N_2_ or He at a pressure of 5.5 bar. A linear rail was used to analyze the QuickStrip spots with the DART in scanning (transmission) mode and a speed of 0.5 mm/s. To help eliminate sample carryover, a sample delay was set to 10 s between each sample as well as a blank spot between each triplicate set. The linear rail passes each spot through the stream of metastable He and the ions formed from the sample are transferred through a ceramic capillary into the mass spectrometer. Each spot takes roughly 10 s at the 0.5 mm/s linear rail speed, and the analysis of the full 12 spots takes just under 6 min. This could be shortened significantly if the sample delay time was shortened or eliminated. High-purity nitrogen and helium served as standby gas and DART ionization gas, respectively.

#### 3.3.3. Data Processing and Analysis Software

All mass spectral and ion mobility data were collected using timsControl and analyzed using Compass DataAnalysis software (both from Bruker, Billerica, MA, USA). Calibration curves and tables were generated using Microsoft Excel. Peak processing parameters used the Sum Peak algorithm and the following parameters: S/N threshold 10; relative intensity threshold 0.1%; absolute intensity threshold 100%; and Background Subtract Analysis Mode set to Xpose with a retention time window of ±0.5 s and a ratio of 5.

## 4. Conclusions

DART-MS, DART-MS/MS, and DART-IM-MS analysis were successfully implemented on PFAS and PPD compounds, proving the ability of this methodology to directly analyze environmentally critical samples at high throughput. The data acquired provided a better understanding of PFAS and PPD ionization under different conditions as well as of the parameters that affect the introduction of analyte into the MS inlet the most. New information about the conformations of gas-phase PFAS and PPD ions was gained. Additionally, the quantification of PFAS and PPD was obtained confidently with minimal sample preparation and high-throughput potential, allowing for a large amount of time to be saved when compared to conventional techniques such as GC–MS or LC–MS, which often require over an hour to conduct. Future studies using this methodology will involve the quantification of other bioaccumulative molecules or environmentally harmful compounds.

## Data Availability

Data are contained within this article and the Supplementary Materials.

## Supplementary Materials

The following supporting information can be downloaded at: https://www.mdpi.com/article/10.3390/molecules30132828/s1, Figure S1: PFAS molecules studied; Figure S2: PPD molecules studied; Figure S3: Background ions; Figure S4: The QuickStrip DART sample holder; Figure S5: The DART-SVP JumpShot ionization source; Figure S6: DART-MS spectrum of tire extract; Figure S7: Mass and mobility calibrants.
