# Supplementary material for: Structural and Quantitative Analysis of Polyfluoroalkyl Substances (PFASs) and Para-Phenylenediamines (PPDs) by Direct Analysis in Real Time Ion Mobility Mass Spectrometry (DART-IM-MS)
Source: Molecules. 2025 Jun 30;30(13):2828. doi: 10.3390/molecules30132828 (PMC12251324; doi:10.3390/molecules30132828)
Supplement: Supplementary file 1 [file molecules-30-02828-s001.zip › molecules-3687576-supplementary.pdf]

molecules

Structural and Quantitative Analysis of Polyfluoroalkyl Substances (PFASs) and Para-Phenylenediamines (PPDs) by Direct Analysis in Real Time Ion Mobility Mass Spectrometry (DART-IM-MS)

Calum Bochenek [1], Jack Edwards [1], Zhibo Liu [2] and Chrys Wesdemiotis [1,2,*]

[1] Department of Chemistry, The University of Akron, Akron, OH 44325, USA; cb332@uakron.edu (C.B.); je94@uakron.edu (J.E.)
[2] School of Polymer Science and Polymer Engineering, The University of Akron, Akron, OH 44325, USA; zl66@uakron.edu
* Correspondence: wesdemiotis@uakron.edu

Supplementary Information

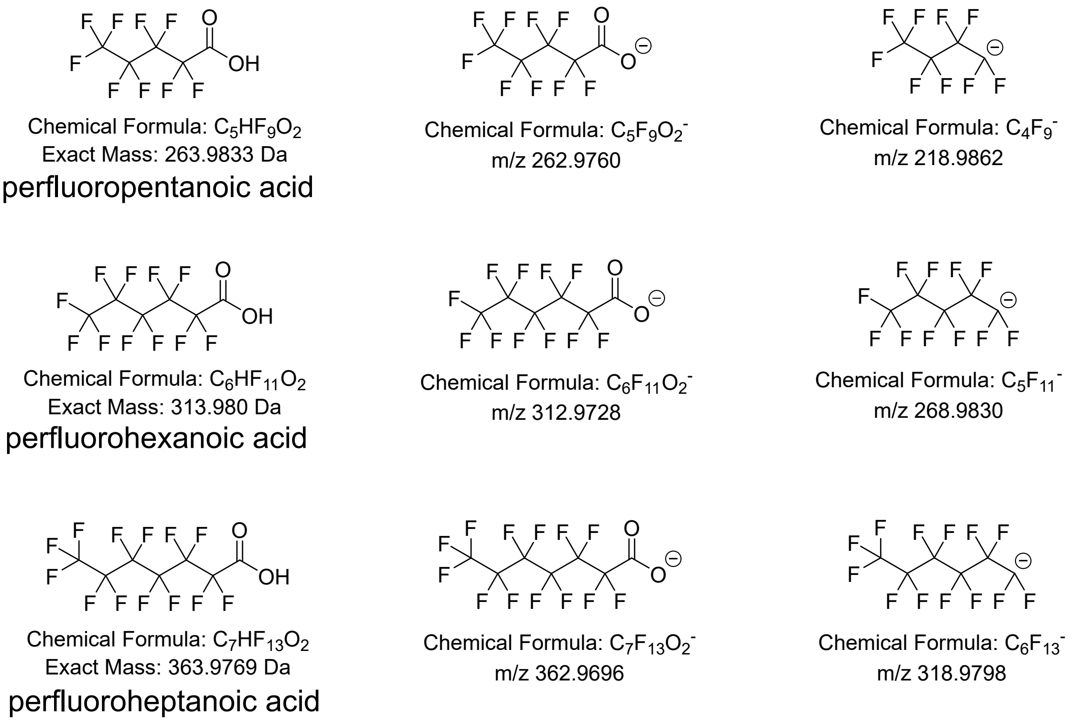

Figure S1: PFAS molecules studied and their $[M – H]^-$ and $[M – CO_2H]^-$anions observed in negative ion mode DART-MS spectra: top, perfluoropentanoic acid (PFPA); center, perfluorohexanoic acid (PFHexA); bottom, perfluoroheptanoic acid (PFHepA). All quoted mass and *m*/*z* data are monoisotopic values.

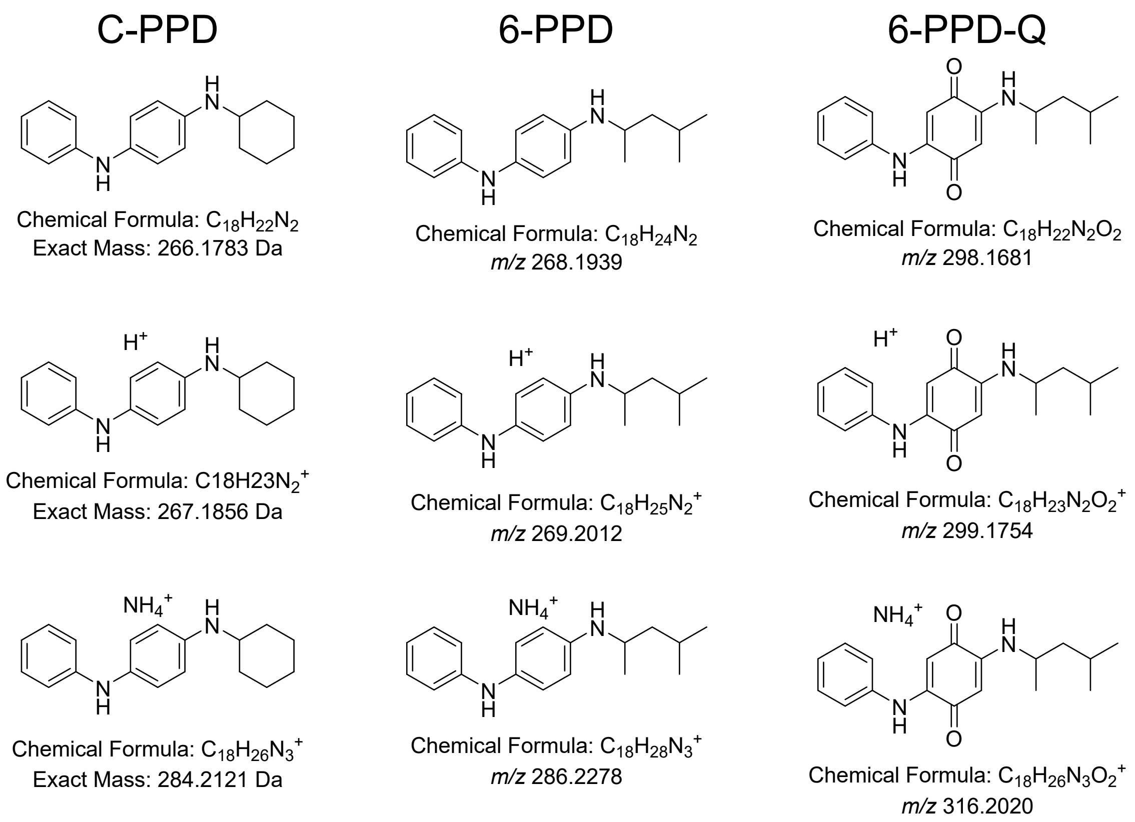

Figure S2: PPD molecules studied and their $[M + H]^+$ and $[M + NH_4]^+$ ions observed in positive ion mode DART-MS spectra: left, N-cyclohexyl-N'-phenyl-p-phenylenediamine (C-PPD); center, N-(1,3-dimethyl)-N'-phenyl-p-phenylenediamine (6-PPD); right, 6-PPD-quinone). All quoted mass and *m*/*z* data are monoisotopic values.

Compound	Ion Structure
Lactic Acid	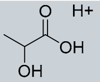 Chemical Formula: $C_3H_7O_3^+$ *m/z* 91.0390
Caprolactam	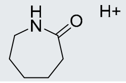 Chemical Formula: $C_6H_{12}NO^+$ *m/z* 114.0913
Myristic Acid	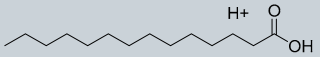 Chemical Formula: $C_{14}H_{29}O_2^+$ *m/z* 229.2162
Palmitic Acid	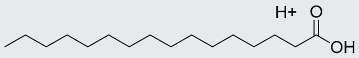 Chemical Formula: $C_{16}H_{33}O_2^+$ *m/z* 257.2475
Oleic Acid	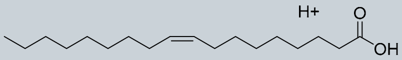 Chemical Formula: $C_{18}H_{35}O_2^+$ *m/z* 283.2632
Stearic Acid	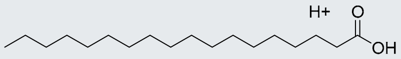 Chemical Formula: $C_{18}H_{37}O_2^+$ *m/z* 285.2788
Cholestadiene	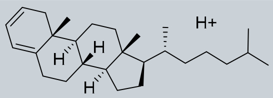 Chemical Formula: $C_{27}H_{45}^+$ *m/z* 369.3516
Bis(ethylhexyl Phthalate)	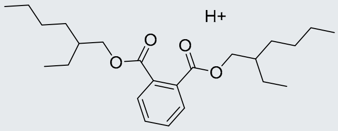 Chemical Formula: $C_{24}H_{39}O_4^+$ *m/z* 391.2843

Figure S3: Identified background ions from adventitious compounds in the atmosphere, which were used as internal calibrants for PFAS and PPD mass analysis. All quoted *m*/*z* data are monoisotopic values. Lactic acid most likely originates from operator perspiration and caprolactam from the Nylon seals within the DART source. The other compounds are common atmospheric contaminants in chemical laboratories.

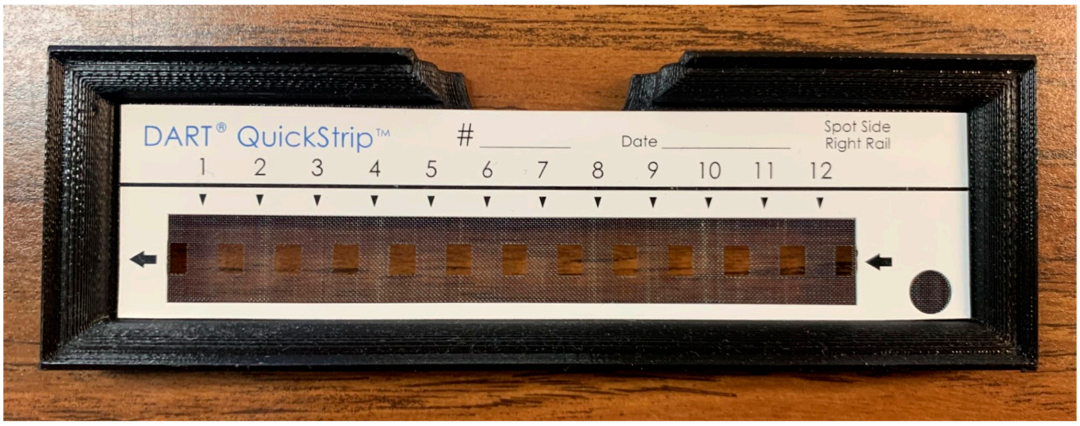

Figure S4: The QuickStrip DART sample plate which can hold up to 12 samples that are successively moved into the DART ion source at a preset speed.

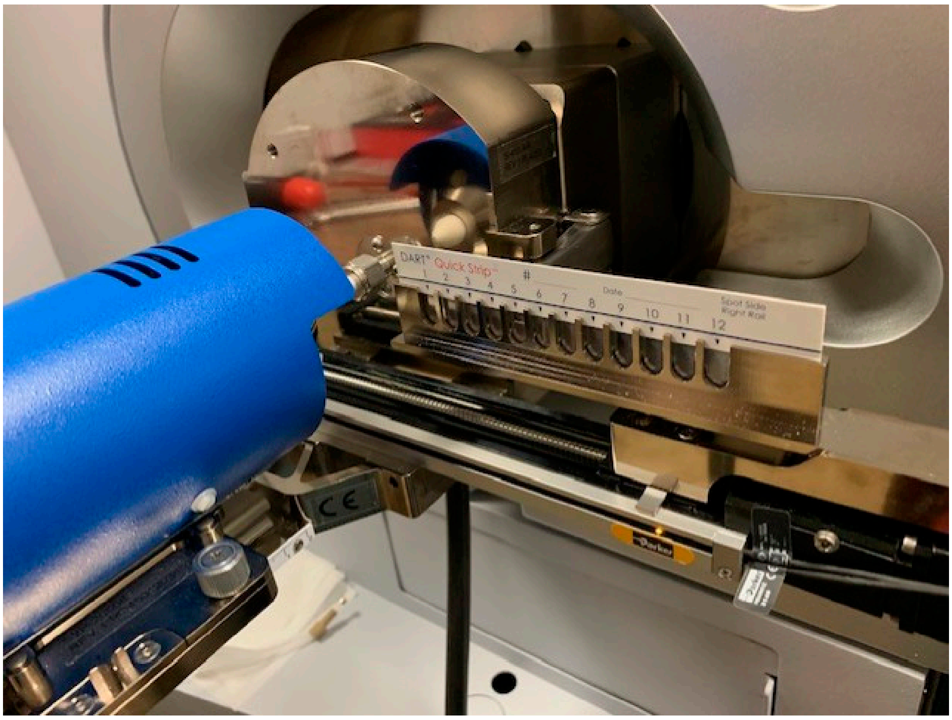

Figure S5: The DART-SVP JumpShot ionization source attached to the timsTOF Pro 2 Bruker Q/ToF mass spectrometer.

DART ionization utilizes heated metastable gas atoms (mainly He) or molecules (mainly N_2) to desorb and ionize target compounds. The metastable particles are produced through plasma generation using a high-voltage needle, which creates both charged and electronically excited neutral (metastable) species. The charged species are removed by electrostatic deflection, and the remaining beam of metastable atoms/molecules is heated before it exits the source and strikes the sample and ambient air. Collisions with the sample lead to desorption of sample molecules, while collisions with air lead to an atmospheric pressure chemical ionization (APCI) plasma that ionizes desorbed sample molecules to positive or negative ions, either of which can be transferred into the mass spectrometer by proper adjustment of the instrument polarity. The exit of the DART ionization source is typically positioned a few millimeters away from the entrance to the mass spectrometer, with samples introduced directly into the open-air ionization region.

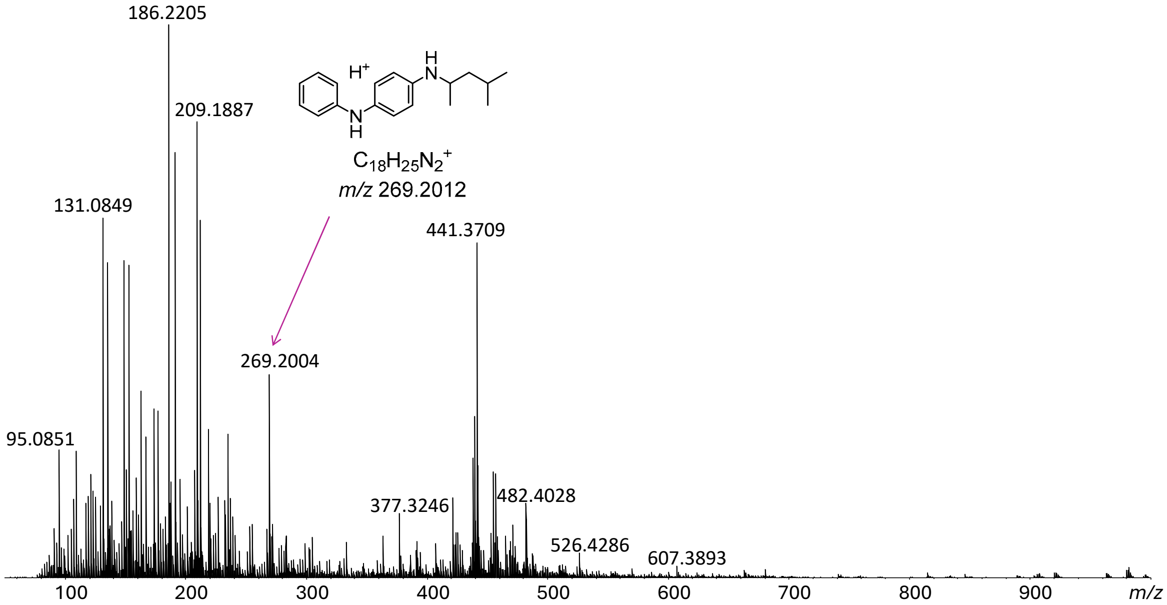

Figure S6: DART-MS spectrum of the THF extracts form a 10 mg piece of a used automobile tire. The tire piece was cut from the tread and soaked in 100 μL of THF for ~7 days. The spectrum includes a peak for the $[M + H]^+$ ion from 6-PPD (*m/z* 269) with an absolute intensity of 15,143 arbitrary units and the CCS of protonated 6-PPD (168.2 Å^2). According to the calibration curve in Figure 2b, this intensity corresponds to a concentration of 43.94 ng/mL.

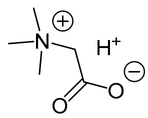

$C_5H_{12}NO_2^+$
m/z 118.08626
$1/K_0$ = 0.5423 m^2/V•s

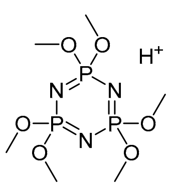

$C_6H_{19}N_3O_6P_3^+$
m/z 322.04812
$1/K_0$ = 0.7319 m^2/V•s

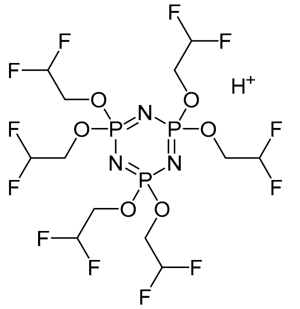

$C_{12}H_{19}F_{12}N_3O_6P_3^+$
m/z 622.02896
$1/K_0$ = 0.9848 m^2/V•s

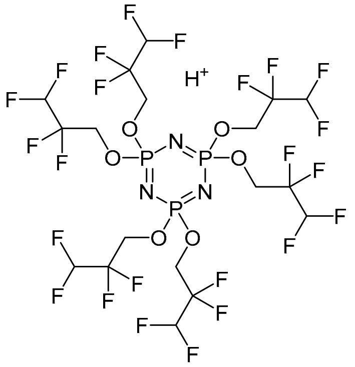

$C_{18}H_{19}F_{24}N_3O_6P_3^+$
m/z 922.00980
$1/K_0$ = 1.1896 m^2/V•s

Figure S7: Agilent tune mix ions used for mass and ion mobility calibration. Molecular formula, mass, and reduced mobility are shown for each ion. All quoted mass and *m*/*z* data are monoisotopic values.
